# Extraosseus Ewing's Sarcoma in Pancreas: A Review

**DOI:** 10.7759/cureus.7505

**Published:** 2020-04-01

**Authors:** Dharti Patel, Nitish Singh Nandu, Aravind Reddy

**Affiliations:** 1 Internal Medicine, Oak Hill Hospital, Brooksville, USA; 2 Internal Medicine, Chicago Medical School, Rosalind Franklin University, North Chicago, USA

**Keywords:** ewing’s sarcoma, pancreas, primitive neuroectodermal tumor, extraosseous

## Abstract

Primitive neuroectodermal tumors (PNET, previously referred to as peripheral neuroepithelioma) are rare malignant tumors with various degrees of differentiation belonging to the Ewing’s family of sarcomas. They are classified as round cell tumors arising from soft tissues. In rare instances, PNETs may arise from solid organs containing neuroendocrine cells of kidney, bladder, heart, lungs, parotid glands and pancreas. Most cases occur in the second decade of life with a slight preponderance in males. PNET of the pancreas is an aggressive tumor with multiple recurrences and a relatively poor prognosis. These tumors should be considered in the differential diagnosis, especially in a diagnosed pancreatic tumor in individuals less than 35 years of age. Due to the nature of the tumor, surgery with subsequent chemoradiation are widely accepted modalities despite the poor prognosis. In this article, we review 25 cases of extraosseous Ewing’s sarcoma (ES) of the pancreas which to the best of our knowledge, enlists most cases reported in the literature thus far.

## Introduction and background

Ewing sarcoma is a poorly differentiated, aggressive, malignant, round cell tumor without cellular or structural differentiation more commonly diagnosed in children younger than ten. It is the second most common malignant bone tumor in children, after osteosarcoma. The incidence of Ewing sarcoma is 1 per million for people of all ages in the United States and has remained unchanged for 30 years [1]. It is most common in Caucasians, and less frequently seen in Asians and African Americans [2,3]

The clinical entity of PNET was identified by Stout in 1918, initially seen in the peripheral nerves [3]. Tefft described the extraosseous form of Ewing’s sarcoma (EES) in 1964. PNETS have also been found in other sites such as the kidney, urinary bladder, uterus, gallbladder, lung, and vagina, and vulva [4-9]. Peripheral primitive neuroectodermal tumors rarely arise in organs and it is extremely uncommon for PNETs to originate in the pancreas. Batsakis et al. divided the PNET family of tumors into the following three groups, based on the origin of the tissue- central nervous system tumors including primitive neuroectodermal tumors in the brain and spinal cord, tumors derived from the autonomic nervous system called neuroblastomas, and tumors originating outside the central and autonomic nervous system called Peripheral primitive neuroectodermal tumors (PPNETs) [4]. 

The ES/PNET family also includes several other related clinicopathologic neoplastic entities such as malignant small-cell tumors of the thoracic pulmonary region (Askin’s tumor, described by Askin in 1979), paravertebral small-cell tumor, atypical ES, PNET of bone, and extraosseous ES. These neoplasms exhibit a neural phenotype, express the MIC2-protein (CD99) and display the same chromosomal translocation t (11; 22) (q24; q12) in about 85% of the cases and hence, can be helpful in diagnosis and have a prognostic value [1,2] 

PNETs usually originate in soft tissues and bone. Luttges, et al. found that only two cases among 600 primary pancreatic neoplasms were pancreatic PNETs [9]. The tumor is of insidious onset and patients usually have no specific clinical symptoms. The most common presentation of an extraosseous tumor is a rapidly growing mass associated with localized pain. The diagnosis of peripheral primitive neuroectodermal tumors of the pancreas encompasses clinical symptoms, pathological characteristics, immunohistochemical features, and molecular genetic analysis. Complete surgical resection followed by radiation and chemotherapy is the currently accepted treatment, although the prognosis remains poor due to the highly aggressive nature of this cancer [7]. Chemotherapeutic regimens that have been used in various combinations include vincristine, cyclophosphamide, actinomycin D and doxorubicin. The prognosis of the extraosseous sites of PNETs is poor due to the delay in diagnosis, and also due to the age of the patient, tumor bulk, location and the highly aggressive nature of the tumor [10] . 

## Review

This literature review talks about Pancreatic Ewing’s Sarcoma (ES/PNET). In total, 25 cases have been reported in the literature to our knowledge. From the review of the 25 cases, we observed that ages ranged from 2 years to 60 years while the average age was around 23 years. No significant sexual predominance was identified in the literature review (13 male patients and 12 female patients). The most common symptoms reported were abdominal pain (68%), jaundice (20%), nausea (16 %), and anemia (16 %). Endocrine disorders such as hyperglycemia and precocious puberty were accompanied in some cases [11] . Ten patients out of 25 had documented chromosomal translocations t(11; 12) (q24; q12). The most common site for peripheral PNET was the head of the pancreas with the size ranging from 3.5 cm to 11 cm.

Abdominal CT and MRI are the two most common modalities to visualize pancreatic tumors, with MRI being the more sensitive imaging study. Tan et al. reported that the radiographic characteristics of the lesion have an isodense or hypodense on unenhanced CT, isointense on T1WI, and can be either isointense or hyperintense on T2WI as revealed by MRI [12]. Pancreatic tumors have ill-defined borders and irregular shapes with heterogeneous enhancement. Due to necrotic areas within the tumor, variable densities have been noticed on the CT scan of the abdomen. Although there is no close relationship with the arteries, some tumors may have intensification in the focal areas on CT in the arterial phase. In the advanced stages of the tumor, invasion into the surrounding organs and metastasis may be seen [13-15]. 

Gene studies have shown that the fusion of the FLI1 gene on chromosome 11 and the ERG gene on chromosome 22 to be associated with PNES. The products of the fusion genes resulting from the translocations are specific to the type of tumors. In reports of ES/PNETs, there are eight cases in which chromosome translocation is t (11;22) (q24;q12) while three cases had t (21;22) (q22; q12). Loss of cosmids F7 and E4 distal of the EWS-R1 breakpoint in nearly all cells was noted in one case [5,10,16-20] .

ES is an undifferentiated tumor lacking neural differentiation in the primitive cells. However, some tumors have cells with neural differentiation in the form of Homer-Wright rosettes. Histologically, there is a presentation of small round blue cells in the form of monotonous sheets with hyperchromatic nuclei and scant cytoplasm [16]. All 25 cases in this review meet these histologic criteria. The tumor consists of extensive necrotic areas with viable tissue usually preserved around blood vessels [18-21]. A known marker for the diagnosis of PNET are p30/32MlC2 [20]. Nonspecific monoclonal antibodies like CD99, O13, HBA71, 12E7, RFB1 were also tested, although none of them are specific for PNETs [21]. Neural markers like Neuron Specific Enolase (NSE), Chromogranin A (CgA), Synaptophysin (Syn) can also be positive [22-24].

There are no definite guidelines for the pathological criteria for the diagnosis of the peripheral PNETs. The combination of clinical signs and symptoms such as abdominal pain with an abdominal mass, jaundice, vomiting, dyspepsia, pathological characteristics like sheets of small round blue cells with hyperchromatic nuclei and scant cytoplasm; immune-histochemical features stained positive for CD99, O13, HBA71, 12E7, RFB1 and neural markers like Neuron Specific Enolase (NSE), Chromogranin A, Synaptophysin; cytogenetic analysis for MIC-2 gene and t [11;22] [q24; q12] suggest a diagnosis of PNETs. Histological features and distinction from other small round cell tumors are the principle criteria for the diagnosis of PNET. In pancreatic PNETs, specific characters of PNET like Homer-Wright (H-W) rosette or atypical rosette array of the cells are rarely present under light microscopy. The important criteria for the diagnosis of ES/PNETs is the cell cytoplasm containing neuronal secretory granules, neurofilaments, and pyknic nucleus granules [25,26]. From our present review, neural markers were positive for NSE (10/25 cases), synaptophysin (2/25 cases) and vimentin (6/25 cases).

Differential diagnoses that are considered based on histomorphology are ES/PNET, desmoplastic small round cell tumor (DSRCT), small cell neuroendocrine carcinoma (SNSC) and pancreatoblastoma. The ES family of tumors consists of small monomorphic round cells histologically, with small nuclei and scant cytoplasm. The same pattern is observed in a large group of tumors. In DSRCT's, which are multicentric tumors, desmoplasia is noticed in the cellular phase which resembles the soft tissue in ES. The tumor cells are positive for Creatine kinase (CK) and desmin [8]. The tumor often stains positive for neuroendocrine markers such as synaptophysin, CD56, and chromogranin. There have been five reported cases of pancreatic PNETs with local recurrence, three cases with lung metastasis and one case with bone metastasis [27-30]. 

Surgical resection with chemoradiation is the widely accepted treatment protocol for PNETs. The commonly used chemotherapeutic agents include doxorubicin, cyclophosphamide, vincristine, actinomycin-D, ifosfamide, and etoposide. A randomized study by Grier et al. compared treatment with three drug regimen (VDC: vincristine, doxorubicin, and cyclophosphamide) versus a five-drug therapy (VDC plus ifosfamide, and etoposide). Subsequently the five drug regimen was established as the gold standard for treatment of PNET’s [20]. Dactinomycin is no longer used in the United States but continues to be used in the Euro-Ewing studies. Increased dose intensity of doxorubicin during the initial months of therapy was associated with an improved outcome in a meta-analysis done prior to the standardization of ifosfamide and etoposide. Radiation therapy has a role in those patients with residual disease after surgery and chemotherapy. Hyperfractionated radiation therapy has not shown to be of any extra benefit; whereas proton-beam radiation therapy and intensity-modulated radiation therapy (IMRT) treatment seem to be promising but lack randomized data.

The main treatment of the disease from our review was surgery along with chemotherapy with or without adjunctive radiotherapy. 18 out of 25 patients received chemotherapy after surgery. Five out of 25 patients had multiple recurrences after combined surgery and chemotherapy.

There is only a little-reported data on the prognosis and survival of these patients who have ES/PNETS. Improvements in the detection of PNETs and their early surgical removal has had a significant impact on the survival rate of these patients. From the literature, it has been observed that patients treated with surgery combined with chemotherapy were alive after 5 years of treatment in 80% of individuals. The 5-year survival rate dropped to 29% in patients with metastasis to the liver or unresectable tumor. Advancing age, advanced stage tumors, non-functioning tumors and those with rapid growth generally have poor outcomes. In patients with advanced PNETs with metastasis, the development of new therapeutic options that arrest tumor growth and progression have shown to be promising [7, 29,30,31,32,33,34]. From our review, 4 out of 25 cases died with the disease within the range of 6 months to 50 months from the time of diagnosis while one died with postoperative complications. The rest were alive with or without disease, with some having multiple metastasis and recurrences. Table 1 summarizes the findings of the 25 cases reviewed.

**Table 1:** Brief review of case reports on PNETs in Pancreas. NA- Not Available; N/P- Not Performed; AWD- Alive with Disease; DWD- Died with Disease; NED- No Evidence of Disease; RT-PCR – Reverse Transcriptase - Polymerase Chain Reaction; VAC- Vincristine, Adriamycin, and Cyclophosphamide; IE- Ifosfamide and Etoposide; VDC- Vincristine, Doxorubicin, Cyclophosphamide.

	Reference	Age/Sex	Symptoms	Physical exam findings	Cytogenetic analysis	Light microscopy and Immunohistochemical(IHC) stain	Diagnosis and Treatment	Metastasis and Recurrence	Clinical follow-up and Outcome
1	Bulchmann et al [6]	6/F	Abominal pain, Anemia,dizziness,	4.0x5.4x3.0 cm	Postmortem FISH demonstrated loss of cosmids F7 and E4 distal of the EWSR1 breakpoint in nearly all cells	Atypical small round cells positive for pancytokeratin, NSE, gamma-enolase and squamoid corpuscles; negative for desmin and chromogranin, focally positive for S100 and MIC2; initial diagnosis pancreatoblastoma; later revised after rosettes found in lymph nodes	Surgery with pancreato-duodenectomy	6 mo; Recurrence	6 months; DOD
2	Mao Y et al [10]	13/F	Dyspepsia, Vomiting	22	NA	NA	Whipple resection, Chemotherapy	NA	NA
3		31/M	Abdominal pain, decreased appetite	NA	NA	NA	Chemotherapy	NA	NA
4		17/M	Abdominal pain	9	t(11;12)(q24;q12)	NA	Radiotherapy, Chemotherapy	N/P	8 months, AWD
5		13/F	Abdominal pain, Diabetes Mellitus type2	3.5	t(11;12)(q24;q12)	Small round and oval cells with scant cytoplasm. The tumor was separated by fibrous connective tissue into the folial parts. Granular nuclear chromatin and karyokinesis phenomenon with unclear nucleoli were found. There were no Homer-Wright rosettes in the tumor cells. positive for CD99, NSE.	Resection of the uncinate process, Radiotherapy / Chemotherapy- Four courses of VAC.	9/36 months, recurrence;12 months, ascites	41 months; AWD
6	Shoustari et al. [34]	47/F	Abdominal pain,abdominal distension,fatigue, weight loss	10 x 15	-	Sheets of small round cells with enlarged round to oval nuclei, fine stippled chromatin, PAS positive clear cytoplasm. Areas of necrosis with focal peritheliomatous proliferation of tumor cells around the blood vessels, increased mitosis, nuclear moulding were noted. In some areas, tumor islands were surrounded by desmoplastic stroma. CD99 positive, while cytokeratin (CK), desmin, synaptophysin (SYP), and chromogranin (CHR) were negative	Excision of the tumor with a distal pancreatectomy and splenectomy,Alternating IE and VAC	Negative	AWD
7	Kumar et al. [27]	20/F	Abdominal pain	11x9	-	Well-circumscribed tumor with a fibrous pseudocapsule composed of sheets of small round cells with enlarged nuclei, fine stippled chromatin, and moderately clear to amphophilic cytoplasm staining periodic acid Schiff stain positive. positive for CD 99, while negative for cytokeratin (CK), insulin, glucagon, synaptophysin (SYP), and chromogranin (CHR).	Whipple resection, VAC along with radiotherapy	bone, liver and lungs	26 months, DOD.
8	Movahedi-Lankarani S et al [5]	17/M	Jaundice, abdominal pain	9	t(11;12)(q24;q12)	Sheets and lobules of small cells with round to oval nuclei and scant cytoplasm, no Homer Wright rosettes, strong membrane positivity for CD99, 5 of 6 cases diffusely expressed cytokeratin AE1/AE3 and 6 of 7 were positive for NSE	Whipple resection, VDC	NA	33 months, NED
9		20/M	Jaundice, abdominal pain	3.5	+ t(11;12)(q24;q12)		Whipple resection, N/P	NA	27months, AWD
10		21/F	Abdominal Pain	NA	t(11;12)(q24;q12)		Whipple resection, NA	NA	Died of post-operative complications
11		25/F	Abdominal Pain	NA	NA		Biopsy, NA	NA	NA
12		25/F	Jaundice, Abdominal pain	8	-		Biopsy, NA	NA	NA
13		13/M	Abdominal pain	6	NA		Biopsy, NA	N/P	43 months; NED
14		6/M	Jaundice, Abdominal Pain	3.5	t(11;12)(q24;q12)		Whipple, VDC	48 months; Recurrence	48 months; DOD
15	Perek et al. [7]	31/M	Abdominalpain, fever	10	-	No lymph node metastases or Homer Wright rosettes, pseudopapillae present. Tumor cells positive for vimentin, CD99, Leu 7 and focally for synaptophysin	Whipple,Radiotherapy, ifosfamide X 6; docetaxel and palliative resection	4months, Recurrence; 24mo/36mo lung	50 months, DOD
16	Welsch et al [22]	33/M	One day history of abdominal pain	18 cm x 18 cm x 6 cm	t(11;12)(q24;q12)	Nests of medium-sized round or oval tumor cells with enlarged round or oval nuclei and scant cytoplasm surrounded by fibrovascular septae; focal Homer Wright rosettes, consistent and strong membranous expression of CD99, strong cytoplasmic staining for vimentin	Laparotomy,Resection, Radiotherapy, 6 cycles of of VIDE, VAI, AST Chemotherapy	Simultaneously, liver, spleen	12 months, AWD
17.	Teixeira et al. [33]	28/F	epigastric pain for 14 days, pruritus, jaundice, choluria, and acholia.	12.8 × 12.1 × 10.9 cm , hardened palpable mass in the epigastric and right hypochondrium regions.	-	small round blue cells with scant cytoplasm arranged in nests with fibrovascular stroma. Few mitosis pictures and several areas of necrosis were also found. Strongly positive for CD99, vimentin, automated CKM (creatine kinase, muscle), and CD56. Negative for chromogranins, synaptophysin, neuroblastoma, myogenin, automated CD10, β-catenin, automated RP (ribosomal protein), and LCA (leucocyte common antigen).	gastroduodenopancreatectomy	-	discharged on the 13th day after surgery, no recurrence
18	Changal et al. [21]	60/M	epigastric Abdominal Pain for 1 month	3 x 3cm lump in the supraumblical region without lymphadenopathy	FISH confirmed t (11; 22) (q24; q12) translocation.	small round cell tumour with pseudorosetting infiltrating the node. Positive for CD99, NSE, FLI-1, synaptophysin and cytoplasmic vimentin. Negative for Cytokeratin (AE1/AE3) and chromogranin, LCA, CD3, CD20, CD79a, CD43, CD34, and TdT were negative.	Biopsy of the peri-pancreatic lymph nodes Received three cycles of VIDE (vincristine, ifosfamide, doxorubicin, and etoposide), planned for a total of 6 cycles	-	A repeat ultrasound after 3 cycles of chemotherapy - tumour shrinked. Prolonged follow up after surgery and reassessment for chemotherapy will be required.
19	Schutte et al. [32]	2/F	Pubic hair, breast development, vaginal bleeding for 6 months and an upper abdominal mass, markedly elevated estrogen levels and a prominent, large uterus	6 x4	NA	Tumor invaded pancreatic surface, but not adjacent structures; resected lymph nodes not involved, but LVI present; examining pathologist’s ‘‘best diagnosis’’ was PNET with divergent differentiation	Distal pancreatomy,Adjuvant chemotherapy with VDC alternating with cisplatin and etoposide	-	hormone levels normalized by 1 month after surgery, CT scans showed NED at 1 year follow-up and all pubertal changes regressed
20	Menon et al. [28]	8/F	Abdominal pain and menstrual bleeding, breast development and pubic hair; markedly elevated estradiol levels	10 X 6 X 10	NA	Mass occupying whole pancreas and obstructing distal CBD found at laparotomy; no lymph node or other metastases; sheets of small round cells, MIC2 positive and LCA negative	Laparotomy with biopsies and cholecystostomy; chemotherapy and radiotherapy; cumulative doxorubicin	-	CR without further surgery, hormone levels normalized and pubertal signs regressed; presented with cardiac failure 1 month after completing treatment, fatal cardiac arrest 19 months after diagnosis
21	Doi et al. [23]	37/M	jaundice	NA	FISH showed an EWSR1 rearrangement at 22q12	Atypical small round cells with scant cytoplasm and round nuclei with distinct nuclear membranes, positive for vimentin, CD99 (MIC2), CD56 and NSE; one lymph node was involved	Pancreato-duodenectomy and hepatic resection,7 cycles of VDC alternating with IE, as well as radiation therapy to bone metastases plus RFA of one hepatic lesion found on FDG-PET/CT after resection	multiple liver and lung metastases; Bone metastasis	One year after diagnosis, lung and bone tumors had diminished; was in good health at time of writing
22	Jing et al [31]	24/F	Exophytic PNET in pancreatic uncinate process	10 X 10 X 8	NA	NA	Surgery, Radiation and chemotherapy for recurrent disease;	Recurrent PNET	Doing well at time of report
23	Bose et al. [24]	35/M	gallstone pancreatitis	3	FISH using a probe for the EWSR1 gene located at 22q12 revealed a rearrangement hybridization signal in each of 100 nuclei analyzed	Small, round and undifferentiated hyperchromatic tumor cells with oval to round nuclei, coarse chromatin and scant cytoplasm arranged in trabeculae, sheets and lobules, strongly and diffusely immunoreactive to vimentin and CD99	Distal pancretectomy splenectomy and cholecystsecomy. Adjuvant VAC alternating with IE (no specific evidence of malignancy seen on postoperative PET/CT)	NA	Doing well at time of writing (18 months from diagnosis) with no evidence of recurrence on PET/CT performed at completion of adjuvant treatment
24	Maxwell et al. [25]	11/M	Fatigue, abdominal pain	9.8 X 7.8 X6.4	EWSR1-ERG fusion transcript by RT-PCR	Biopsies from duodenal ulcer showed a small blue cell tumor with strong diffuse membranous staining for CD99; also positive for broad-spectrum cytokeratin and vimentin	Whipple procedure, VDC alternating with IE	NA	CT after 3 months of chemotherapy showed significant shrinkage of mass and LAD; EGD showed resolution of ulcer
25	Saif et al [29]	38 /F	Abdominal pain , epigasrtic tenderness	8x10 cm	t(11;22) (q24;Q12).	Sheets of small round cells with enlarged round to oval nuclei, fine stippled chromatin, moderately clear to amphophilic cytoplasm, Periodic acid schoff +ve, CD99 +ve , ,	Distal pancreatectomy with splenectomy. 2 cycles of ifosfamide, etoposide. And VAC ( Vincristine, adriamycin, cyclophosphamide )	No metastatis or recurrence reported	Six month follow up after adjuvant chemo, no evidence of cancer

## Conclusions

PNET is an aggressive malignant tumor with unavoidable recurrences. Our review of 25 reported cases of PNET till date indicates that from the lack of concrete diagnostic pathological criteria, it is easy to misdiagnose PNET. In cases of pancreatic tumors, it is necessary to highlight the importance of considering ES/PNET early on in the differential diagnosis for improved prognosis and better longevity.
